# Novel BC02 Compound Adjuvant Enhances Adaptive and Innate Immunity Induced by Recombinant Glycoprotein E of Varicella-Zoster Virus

**DOI:** 10.3390/vaccines10122155

**Published:** 2022-12-15

**Authors:** Junli Li, Lili Fu, Xiaonan Guo, Yang Yang, Jiaxin Dong, Guozhi Wang, Aihua Zhao

**Affiliations:** 1 Division of Tuberculosis Vaccine and Allergen Products, Institute of Biological Product Control, National Institutes for Food and Drug Control, Beijing 102629, China; 2 Key Laboratory for Quality Research and Evaluation of Biological Products, National Medical Products Administration (NMPA), Beijing 102629, China; 3 Key Laboratory of Research on Quality and Standardization of Biotech Products, National Health Commission (NHC), Beijing 102629, China; 4 National Engineering Laboratory for AIDS Vaccine, School of Life Sciences, Jilin University, Changchun 130012, China; 5 School of Life Science and Biopharmaceuticals, Shenyang Pharmaceutical University, Shenyang 110016, China

**Keywords:** varicella-zoster virus, glycoprotein E, BC02 compound adjuvant, innate immunity

## Abstract

Both adaptive and innate immunity responses are necessary for the efficient elimination of different pathogens. However, the magnitude, quality and desired type of immune response specific to the co-administered antigen is largely determined by adjuvants. BC02 (BCG CpG DNA compound adjuvants system 02) is a novel compound adjuvant with independent intellectual properties, which is composed of BCG CpG DNA biological adjuvant with Al(OH)_3_ inorganic salt adjuvant acting as a delivery system. Its safety and strong adjuvant efficacy have been effectively verified in preclinical and clinical trials (Phase Ib, ClinicalTrials.gov Identifier: NCT04239313 and Phase II, ClinicalTrials.gov Identifier: NCT05284812). In this study, we report the level of cell-mediated immunity (CMI) and humoral immune response induced by the BC02 novel adjuvant combined with different doses of varicella-zoster virus (VZV) glycoprotein E (gE) in a mouse model. In addition, we conducted preliminary in vitro experiments to explore the enhancement of RAW264.7 cell immune activity by BC02 adjuvanted-gE experimental vaccine to activate innate immune response. The results showed that the BC02-adjuvanted low, medium or high dose of gE were highly effective in eliciting both CMI and humoral immune responses to the immunized mice, respectively. The production of gE-specific IFN-γ and IL-2-specific T cells was established within 28 days after booster immunization. In particular, the effect of BC02-adjuvanted medium dose of gE has been shown to be more prominent. Meanwhile, fluorescent antibody to membrane antigen (FAMA) and serum antibody plaque reduction tests have also shown that the BC02 adjuvanted-medium dose of gE antigen could induce the secretion of neutralizing antibodies against clinically isolated VZV strains in mice. In addition, our findings have shown that 1/25 dose of gE+BC02 medium dose experimental vaccine can significantly induce the secretion of innate immune cytokines TNF-A, MCP-1, IL-6 and GM-CSF and up-regulate the costimulatory molecules CD40, CD80 and I-A/I-E on RAW264.7 cells; and it has also been activated to form M2 macrophages. At the same time, RAW264.7 cells were stimulated for 12 h, and their phagocytosis was significantly enhanced. Taken together, these results suggest that the BC02 compound adjuvant offers a strategy to induce an appropriate innate and adaptive immunity against the different doses of the VZV gE protein to improve subunit vaccine efficacy, and BC02 may be a promising adjuvant candidate for subunit HZ vaccines.

## 1. Introduction

Vaccines are active immune preparations produced by artificially attenuated, inactivated, or genetically modified pathogenic microorganisms and their metabolites to be used in the prevention from infectious diseases. Vaccines usually consist of two main components: antigen and adjuvant. The antigen is usually a protein, polysaccharide or other compound derived from the pathogen, and the inoculated body produces an adaptive immune response to it. The adjuvant, on the other hand, enhances the strength and longevity of immune responses and may also influence the type of immunity, reduce the used dose of vaccine antigens and number of immunizations, increase the formation rate of the initial immune response, and slow down the depletion rate of vaccine antigens. Adjuvants, such as aluminum salt-based adjuvants (alum), were conventionally designed to form a depot of antigen at the injection site to allow its controlled release, mimicking local infection [1]. However, it is now known that they are able to trigger innate immunity by interacting with antigen-presenting cells (APCs) in order to direct the adaptive immune system to produce specific and effective immune responses against the antigen [2,3].

Cytosine phosphate guanosine (CpG) oligodeoxynucleotides (ODN) are short, single-stranded synthetic DNA molecules. Their motifs are often observed in bacterial and viral DNA and recognized by Toll-like receptors 9 [4,5]. Since the FDA approval of the Dynavax Technologies CpG ODN 1018 (5′-TGACTGTGAACGTTCGAGATGA-3′) adjuvanted-HBsAg HBV vaccine HEPLISAV-B™ in November 2017 [6,7], Toll-like receptor agonists have rapidly become one of the hot topics in the development of vaccine adjuvants. CpG ODN, the main agonist of Toll-like receptor (TLR) 9, is currently used as one of the adjuvant components in several COVID-19 vaccines under Phase I or Phase II clinical trials, such as MVC-COV1901 [8,9], SCB-2019 [10] and SpikoGen^®^ [11]. The BC02 (BCG CpG DNA compound adjuvants system 02) is a compound adjuvant consisting of the Al(OH)_3_ inorganic salt adjuvant plus BCG CpG DNA (Unmethylated CpG motif-containing DNA fragment from the genome of BCG). It has been shown by our previous studies to have the advantage of integrating all components while simultaneously inducing the Th1 and Th2 immune responses [12,13]. In addition, a genome-wide microarray analysis found that each component had a synergistic enhancement in vivo and in vitro in activating macrophages to participate in innate immune responses [14,15].

The magnitude and quality of the adaptive immune response could be affected by different adjuvant formulations to achieve optimized protection against specific pathogens. Similarly, the immune stimulatory effect of the same adjuvant formulation is quite different when combined with different types or doses of antigen. The AEC/BC02 vaccine combined with isoniazid and rifamentine can significantly reduce the organ lesions and viable bacterial load in spleen and lung while improving the therapeutic effect on *Mycobacterium tuberculosis* infected guinea pigs and mice. At the same time, the risk of the Koch phenomenon, induced by the recombinant AEC/BC02 vaccine, is significantly reduced [16,17,18], with an irreplaceable role of the BC02 adjuvant.

However, the adjuvant performance of BC02 combined with varicella-zoster virus (VZV) at different doses of glycoprotein E (gE) has to be investigated, especially in terms of innate immune initiation. To this end, in this study, different doses of VZV gE protein were used as antigen-compatible BC02 compound adjuvant or Al(OH)_3_ adjuvant to explore the effects of the new compound adjuvant on the cellular immunity and humoral immunogenicity of the gE protein in the mouse model. We further verified whether BC02 combined with gE has the same good innate immune priming effect at the cellular level in vitro. This work provides experimental evidence for BC02 as a possible adjuvant of the VZV gE protein vaccine.

## 2. Materials and Methods

### 2.1. Animal Ethics Consideration

All animal studies were carried out at the Institute for Laboratory Animal Resources of the National Institute of Food and Drug Control (NIFDC), and all experiments involving laboratory animals were approved by the Institutional Animal Care and Use Committee (IACUC) of NIFDC, Approval Code: 2021(B)036. Healthy mice were maintained in specific-pathogen-free (SPF) conditions and housed in a temperature- and humidity-controlled chamber with a 12 h light/dark cycle. They were acclimated to the environment for three days before immunization, had ad libitum access to food and water, and were cared for by specialized staff throughout.

### 2.2. Materials and Reagents

The BC01 novel biological adjuvant was developed and preserved in our laboratory. Alhydrogel^®^ aluminum hydroxide gel adjuvant and CellMask™ Deep Red plasma membrane stain were purchased from InvivoGen, USA. IFN-γ and IL-2 ELISPOT kits were purchased from Mabtech, Sweden. Mouse TNF-α, MCP-1, IL-6 and GM-CSF Cytokine ELISA MAX^TM^ Deluxe Kit, anti-mouse PE-Cy7-CD40 (Clone: 3/23), FITC-CD80 (Clone: 16-10A1), PE-CD206 (Clone: C068C2), and APC-I-A/I-E (Clone: M5/114.15.2) fluorescent antibodies were purchased from BioLegend, USA. FlouroSpheres^TM^ Carboxylate-Modified Microspheres were purchased from Thermo Scientific™, USA. Shingrix^®^, an HZ subunit vaccine adjuvanted with AS01_B_ (lot no. AVZVA031B, AA1BA009), was preserved in our laboratory. The recombinant VZV gE glycoprotein, MRC-5 cell line (ATCC: CCL-171) and clinically isolated strains of VZV (Confirmed by sequencing) were provided by Anhui Longcom Biologic Pharmacy Co., Ltd., China. The peptide pool of gE glycoproteins were synthesized by Suzhou Qiangyao Biotechnology Co., Ltd., China. Phosphate-buffered saline (PBS), RPMI 1640 medium, Minimum Eagle’s Medium, fetal bovine serum (FBS) and 100× Penicillin-Streptomycin solution were purchased from Gibco, USA. Anti-VZV monoclonal antibody (IgG2b mAb, 1 mg/mL, soluble in 0.02M PB and 0.25M NaCl (pH 7.6), containing 0.1% sodium azide) and FITC-goat anti-mouse IgG (H + L) were obtained from Merk, USA and Beyotime Biotechnology, China, respectively.

### 2.3. Experimental Vaccine Preparation

To prepare experimental vaccines with BC02 adjuvants and different concentrations of gE antigen, 12.5 µg/mL, 25 µg/mL and 50 µg/mL gE glycoproteins were mixed with 50 µg/mL novel biological adjuvant BC01 and 62.5 µg/mL aluminum hydroxide adjuvant. For the unadjuvanted gE experimental vaccine, 5000 µg/mL endotoxin-free gE protein stock of known molecular weight was diluted with sterile 1 × PBS to 25 µg/mL protein suspension. All vaccine formulations were lightly mixed and placed in a 4 °C thermostatic container for immediate use in mouse immunization.

### 2.4. Immunization and Anatomy of Mice

**Figure 1:**
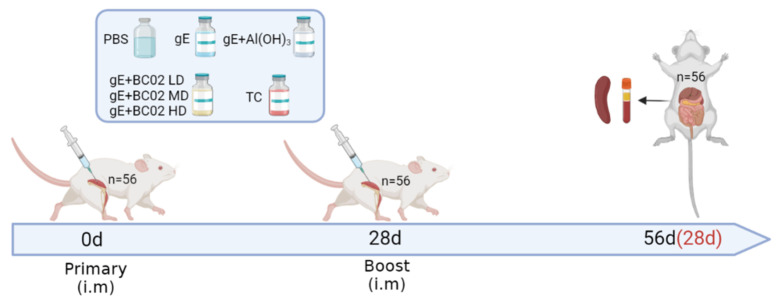
Mouse immunization and sampling schedule. Fifty-six mice were randomly divided into seven groups, and booster immunizations were performed at intervals of 4 weeks after the primary immunization. Mice were sacrificed at 56 days after the primary immunization (28 days after booster immunization) and peripheral blood and spleen was collected. The spleen lymphocytes were isolated for immunological detection. LD: Low dose, MD: Medium dose, HD: High dose.

Six to eight week old SPF female BALB/c mice (20–22 g) were randomly assigned into seven groups with 8 mice in each group, and intramuscular-injection (i.m) immunization was administered twice at a 4-week interval. Vaccines were administered to the different groups as follows: unadjuvanted gE experimental vaccine (5 µg); BC02-adjuvanted gE experimental vaccine with different doses (2.5 µg in low dose, 5 µg in medium dose, 10 µg in high dose); Al(OH)_3_-adjuvanted gE experimental vaccine (5 µg) or PBS in a total volume of 200 µL. Shingrix^®^ was reconstituted by combining gE antigen with AS01_B_ adjuvant according to the manufacturer’s instructions and 1/10 human dose (50 µL) was adjusted to 200 µL with 1 × PBS. Mice were sacrificed at 56 days after the primary immunization (28 days after booster immunization) and peripheral blood and spleen were collected (Figure 1). Peripheral blood was allowed to clot at 4 °C for 6 h before centrifugation at 1200× *g* for 10 min at room temperature. Sera were divided into aliquots for storage at −80 °C. Spleens were removed by aseptic dissection and splenic lymphocytes were isolated by density gradient centrifugation after gentle trituration and immunological analyses were conducted immediately.

### 2.5. gE-Specific IFN-γ and IL-2 ELISPOT Assays

The numbers of immunized mice splenocytes producing IFN-γ and IL-2 were determined by enzyme-linked immunospot (ELISPOT) assays, according to the manufacturer’s protocol. In brief, 1.0 × 10^7^ cells/mL single-cell suspensions were prepared from the spleens of immunized mice and 50 µL cells were seeded in duplicate in ELISPOT plates coated with capture antibodies. Cells were stimulated with gE glycoprotein polypeptide (0.625 µg/mL), concanavalin A (0.1 µg, positive control) or culture media (negative control) for 48 h at 37 °C under a 5% CO_2_ atmosphere. IFN-γ and IL-2-secreting cells were detected using an ELISPOT kit and positive spots were counted using a CTL-ImmunoSpot^®^ S5 UV Micro Analyzer (Cellular Technology Ltd., USA).

### 2.6. VZV Fluorescent Antibody to Membrane Antigen (FAMA) Assays

The clinically isolated VZV strains were co-incubated with MRC-5 cells at 37 °C in a 5% CO_2_ incubator for 4–5 days, and cells were collected when the infection rate reached 50–75%. Infected cells were counted and diluted into a suspension of 1.2 × 10^7^ cells/mL. A total of 10 µL (about 12,000 cells) of single-cell suspension was added to each well of a multi-well slide and placed in a wet box at 37 °C for 40 min to allow the water to completely evaporate, and infected cells were adsorbed in the micropores. Infected cells were fixed with 80% acetone for 15 min, and 10 µL of a 2-fold serial dilution (1:2–1:512) of tested serum was added dropwise to the corresponding wells. Negative (PBS), positive (Anti-VZV mAb, 1:5000) and serum (No FITC-IgG secondary antibodies were added) control wells were set according to the above method and incubated in a wet box at 4 °C for 12 h. Residual serum was removed and the slides were washed 3 times with 1 × PBS for 5 min each time. FITC fluorescently labeled secondary antibodies were diluted 1:100 in PBS, containing a final concentration of 0.01% Evans blue staining solution, and 10 µL was added to each well. After incubation at 37 °C for 1 h in a wet box, slides were washed with 1 × PBS as above. A 3 µL volume of 60% glycerol was added dropwise to each well and a coverslip was applied to enable photography under a fluorescence microscope.

### 2.7. Determination of VZV Neutralizing Antibody Titers by Plaque Reduction Test

The clinically isolated VZV strain (1000 PFU/mL) was mixed with equal volumes of serum to be tested, and incubated at 37 °C for 1 h for virus neutralization. 100 μL serum-virus neutralization solution was uniformly added to a 6-well cell culture plate and incubated at 37 °C and 5% CO_2_ for 1 h. During this period, the virus was evenly adsorbed to the dense monolayer of MRC-5 cells by mixing it every 15 min. At the end of adsorption, 3 mL virus culture medium was added to each well and incubated for 7–10 days to allow the formation of virus plaques. To calculate the percentage of plaque reduction, cell control wells (virus dilution), serum control wells (serum to be tested) and virus control wells (diluted VZV) were prepared as control groups. The formation of plaque was observed and recorded regularly, the protovirus culture liquid was removed, and each well was washed with 1 mL 1 × PBS and 1 mL 0.25% Coomassie brilliant blue R250 staining solution added before incubation for 15 min at room temperature. The remaining dye was rinsed gently with running water and the number of cytopathic plaques was counted.

### 2.8. Cell Culture Supernatant ELISA Assay

**Figure 2:**
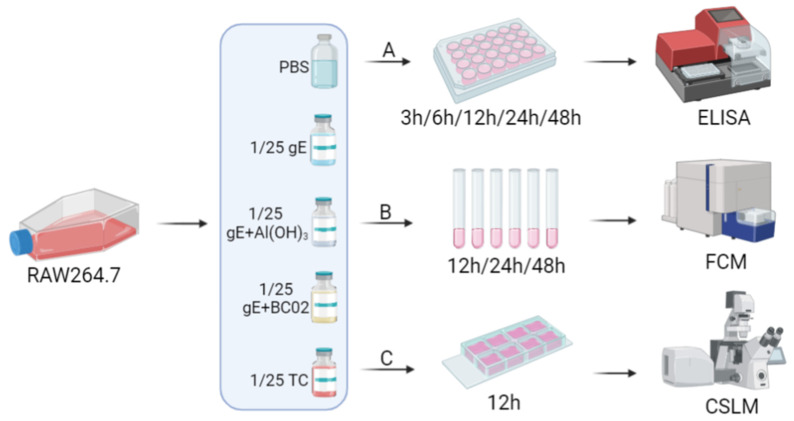
In vitro stimulation analysis of RAW264.7 cells. RAW264.7 cells were stimulated for different times at 37 °C with 1/25 dose of experimental vaccines. (**A**): ELISA was used to detect TNF-a, MCP-1, IL-6 and GM-CSF in cell culture supernatant. (**B**): Flow cytometry was used to detect CD40, CD80, CD206, and I-A/I-E cell surface costimulatory molecules. (**C**): Confocal microscopy was used to detect changes in phagocytosis of RAW264.7. ELISA: Enzyme linked immunosorbent assay, FCM: Flow cytometry, CSLM: Confocal scanning light microscopy.

A Cytokine ELISA MAX^TM^ Deluxe Kit was used to measure the cytokine levels, and the experiments were performed as per the manufacturer’s instructions. In brief, in these in vitro stimulation assays (Figure 2A), 1 × 10^6^ cells/well RAW264.7 cells were stimulated for 3 h, 6 h, 12 h, 24 h and 28 h at 37 °C with 1/25 dose of unadjuvanted gE experimental vaccine, gE + Al(OH)_3_, gE + BC02 MD or TC, respectively. Cell culture supernatants were collected after incubation with the stimulant compounds mentioned above at different time points, and TNF-α, MCP-1, IL-6 and GM-CSF levels were measured using the double antibody sandwich ELISA method.

### 2.9. Cell Surface Costimulatory Molecules Flow Cytometry Assay

RAW264.7 cells (1 × 10^6^ cells) were incubated with 1/25 dose of unadjuvanted gE experimental vaccine, gE + Al(OH)_3_, gE + BC02 MD for 12 h, 24 h, or 48 h, respectively (Figure 2B). The cells were harvested and washed with sterile 1 × PBS 3 times, and were incubated at room temperature with PE-Cy7-conjugated anti-mouse CD40, FITC-conjugated antimouse CD80, PE-conjugated anti-mouse CD206, and APC-conjugated antimouse I-A/I-E for 30 min in the dark. Finally, the cells were fixed with 1% paraformaldehyde and were analyzed using a FACScan flow cytometer. All of the fluorescent antibodies were purchased from Biolegend, USA.

### 2.10. Cell Phagocytosis Confocal Microscopy Assay

For laser scanning confocal microscopy (Figure 2C), 1 × 10^5^ cells/well of RAW264.7 cells were seeded into the 8-well Nunc™ Lab-Tek™ Chamber Slide™ (Thermo Scientific™). After 12 h pre-treatment with 1/25 dose of unadjuvanted gE experimental vaccine, gE + Al(OH)_3_, gE + BC02 MD, the cells were incubated with 1 × 10^4^ FlouroSpheres^TM^ Carboxylate-Modified Microspheres for 1 h in a cell incubator (37 °C, 5% CO_2_). The cells were then labeled with Cell Mask Deep Red plasma membrane stain at 37 °C for 30 min. Finally, the cells were blocked with an anti-fluorescence quenching agent (DAPI) and observed using a Nikon C2 Plus confocal microscope system at ×20 objective, and each experiment was repeated at least three times independently.

### 2.11. Statistical Analysis

Statistical analyses were performed using GraphPad Prism^®^ 8.0 (GraphPad Software, USA). Differences among experimental groups were analyzed using one-way ANOVA with a Tukey’s multiple comparison test. Means and standard errors are expressed as Mean ± S.E. For all analyses, *p*-values of <0.05 was considered statistically significant.

## 3. Results

### 3.1. Higher Induction of gE Specific IFN-γ and IL-2 through the Experimental Vaccine with BC02

**Figure 3:**
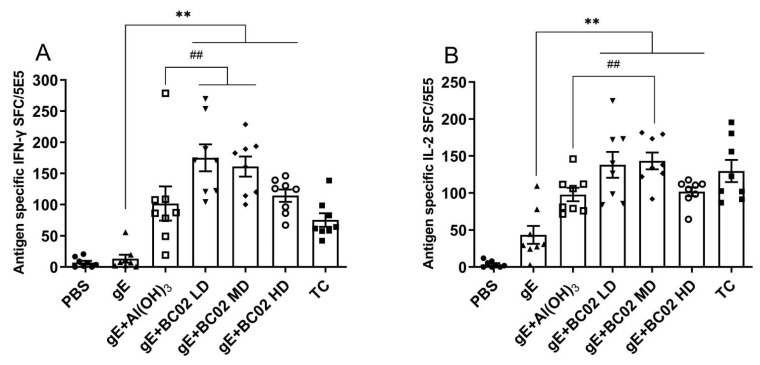
BC02-adjuvanted experimental vaccine induces gE specific cellular immune response. (**A**,**B**): The average number of gE-specific IFN-γ and IL-2 spots forming cells (SFC) in differently immunized mice at 56 days post immunization determined by ELISPOT. Eight mice per immunization group. ** and ^##^ indicates that there is a significant statistical difference between gE + BC02 LD/MD/HD compared with gE, or gE + Al(OH)_3_, respectively, *p* < 0.05. LD: Low dose, MD: Medium dose, HD: High dose.

Our findings showed that the number of antigen-specific IFN-γ cells induced by BC02 combined with low, medium and high doses of gE was significantly higher than that of the non-adjuvanted gE immunized control. The number of spot-forming cells (SFC) of the BC02 adjuvant was 175 ± 22 in the low-dose gE group (LD), 164 ± 16 in the medium-dose gE group (MD) and 114 ± 10 in the high-dose gE group (HD), which were significantly higher than 13 ± 6 in the non-adjuvanted gE experimental vaccine control (all *p* < 0.05, Figure 3A). Moreover, compared with the Al(OH)_3_-adjuvanted gE experimental vaccine, LD and MD were significantly different (*p* = 0.0097 and *p* = 0.0498, respectively, Figure 3A), unlike HD (*p* = 0.9841, Figure 3A).

The number of cells secreting gE antigen specific IL-2 in the spleen lymphocytes of immunized mice was also shown to increase, and SFC in the BC02-adjuvanted gE experimental vaccine group was 138 ± 18 at LD, 143 ± 11 at MD and 102 ± 6 at HD after booster immunization, which were significantly higher than those of the PBS 4 ± 2 and non-adjuvanted gE experimental vaccine group 43 ± 12 (all *p* < 0.05, Figure 3B). Similarly, compared with the Al(OH)_3_-adjuvanted gE experimental vaccine, only MD was significantly different (*p* = 0.0284, Figure 3B), while LD and BH were not (*p* = 0.0583 and *p* = 0.9916, Figure 3B).

### 3.2. Efficient Induction of Anti-VZV Antibody by the Experimental Vaccine with BC02

**Figure 4:**
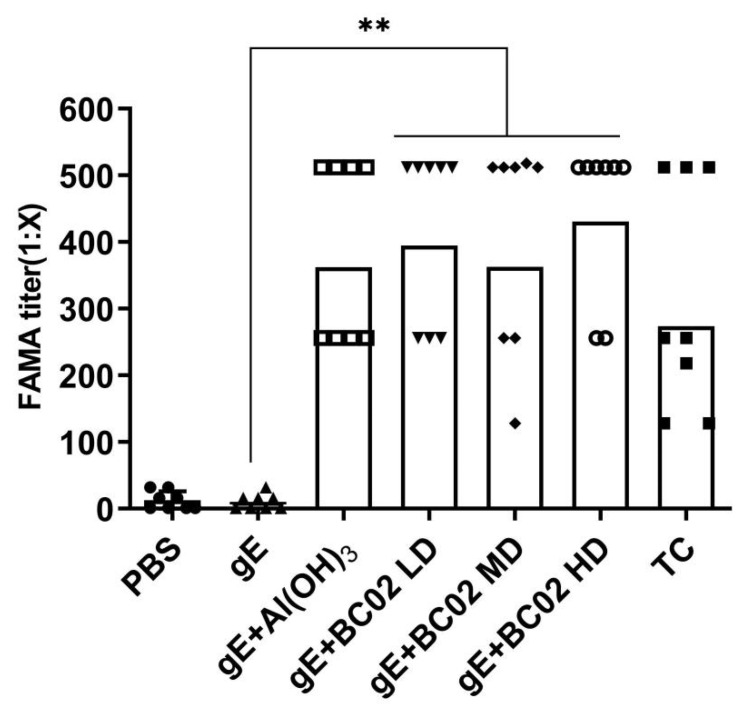
Anti-VZV antibodies induced by BC02-adjuvanted gE experimental vaccine. FAMA serology test to determine susceptibility to VZV infection. Serum from each mouse was diluted at twofold serial dilution (1:2–1:512) to determine the maximum dilution ratio for obtaining the lowest positive fluorescence signal. ** indicates that there is a significant statistical difference between gE + BC02 LD/MD/HD compared with gE, or gE + Al(OH)_3_, respectively, *p* < 0.05. LD: Low dose, MD: Medium dose, HD: High dose.

We found the serum dilution ratio of positive fluorescence signal in the negative control group (PBS) to be significantly lower than that in the test control (TC) (*p* < 0.05, Figure 4), which indicates that the whole test system was established. Under this test system, the FAMA antibody level induced by BC02 adjuvanted with low, medium and high gE was much higher than that of gE without adjuvanted BC02. The dilution ratio of the antibody positive fluorescence signal was 416 ± 47 in LD, 401 ± 57 in MD and 448 ± 42 in HD, which was significantly higher than that of the non-adjuvanted gE experimental vaccine control (*p* < 0.05, Figure 4). However, the FAMA positive fluorescence signal of BC02 combined with low, medium and high doses of gE was basically the same among the three groups, although the BH was slightly higher. However, the difference among the three groups was not statistically significant (*p* > 0.05, Figure 4), which does not indicate a dose-dependent manner of FAMA positive fluorescence signal antibody level with gE.

### 3.3. Efficient Induction of VZV Neutralizing Antibody through the Experimental Vaccine with BC02

**Figure 5:**
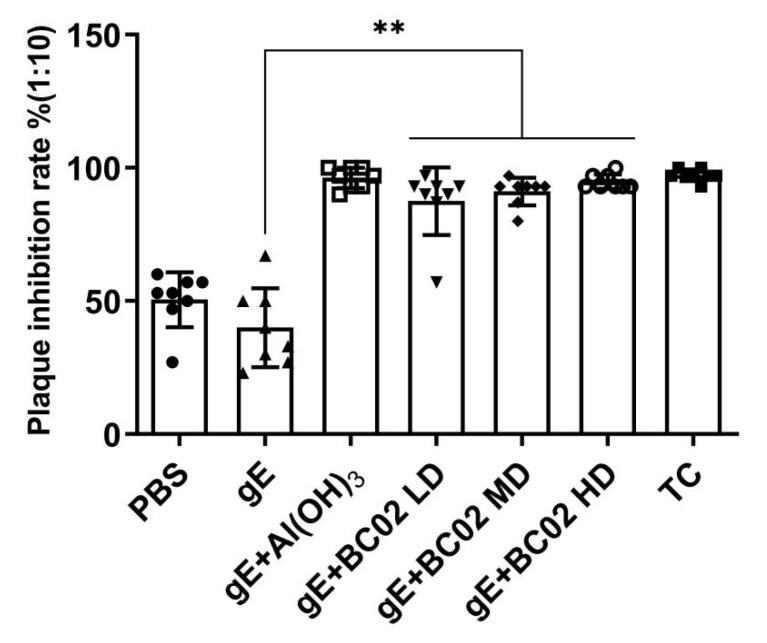
Inhibitory rate of VZV plaque formation by serum antibodies from immunized mice after booster immunization. The immunized mouse serum was diluted at 1:10, and the change of VZV plaque formation inhibition rate in different immunization groups. ** Indicates that there is a significant statistical difference between gE + BC02 LD/MD/HD compared with gE, respectively; *p* < 0.05. LD: Low dose, MD: Medium dose, HD: High dose.

As shown in Figure 5, the analysis of the neutralizing ability of immunized mice serum antibodies against the clinical isolates strain VZV, the plaque inhibition rate of PBS group and the non-adjuvanted gE experimental vaccine group were lower than 50% at 64 days after primary immunization. However, the inhibition rates of VZV plaque formation were (88 ± 4)% at LD, (91 ± 2)% at MD and (95 ± 1)% at HD in the BC02-adjuvanted gE experimental vaccine group. Meanwhile, LD, MD and HD showed a tendency to increase the plaque inhibition rate as the gE dose increased, but the increase in each group and differences among the three groups were not statistically significant (*p* > 0.05, Figure 5).

### 3.4. Higher Induction Cytokines of TNF-α, MCP-1, IL-6 and GM-CSF in RAW264.7 by the Experimental Vaccine with BC02

**Figure 6:**
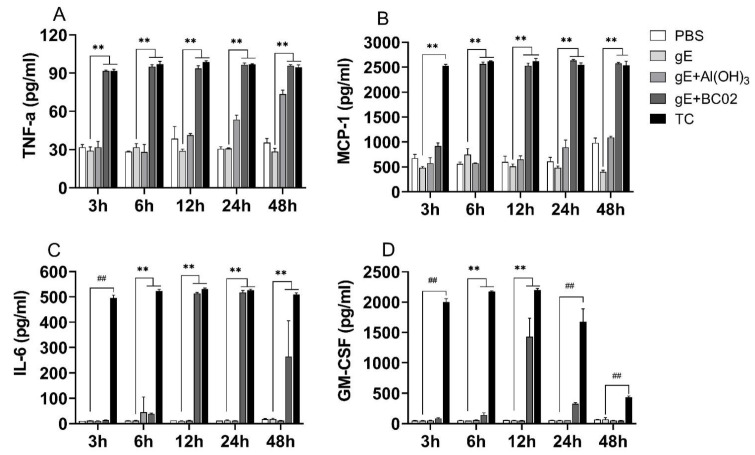
BC02-adjuvanted gE experimental vaccine stimulation induces secretion of cytokines by murine macrophages. (**A**–**D**): RAW264.7 cells were stimulated with 1/25 dose of experimental vaccine for different time points (3–48 h). Cell culture supernatants were collected, and the amount of TNF-a, MCP-1, IL-6 and GM-CSF levels were measured using ELISA. ** and ^##^ indicates that there is a significant statistical difference between 1/25 dose of TC and gE + BC02 MD compared with gE group, or 1/25 dose of TC compared with the gE group, respectively, *p* < 0.05.

When stimulated with 1/25 dose of the MD experimental vaccine, TNF-α and MCP-1 were significantly expressed at different time points. Similar to TC, higher secretion levels of TNF-α and MCP-1 could be observed within 3 h of stimulation, and this increase continued until 48 h, which was significantly higher than that of non-adjuvanted gE experimental vaccine group (all *p* < 0.05, Figure 6A,B). In the 1/25 dose of the Al(OH)_3_-adjuvanted gE experimental vaccine group, the secretion levels of TNF-α and MCP-1 gradually increased with the increase of the stimulation time, but the expression levels remained lower than those of 1/25 MD and TC.

IL-6 and GM-CSF cytokines, which are related to innate immunity, were also significantly expressed at different time points when stimulated with 1/25 dose of TC, showing a statistically significant increase compared with that of the non-adjuvanted gE experimental vaccine group (all *p* < 0.05, Figure 6C,D). However, IL-6 and GM-CSF increased first and then gradually decreased in the 1/25 dose of the MD group; they peaked at about 12 h after stimulation and then began to decrease. In addition, GM-CSF decreased to baseline levels at 48 h of stimulation. It should be noted that it is difficult to stimulate the secretion of IL-6 and GM-CSF with 1/25 dose of the Al(OH)_3_-adjuvanted gE experimental vaccine (Figure 6C,D).

### 3.5. Higher Induction of Cell-Surface Costimulatory Molecules in RAW264.7 by the Experimental Vaccine with BC02

**Figure 7:**
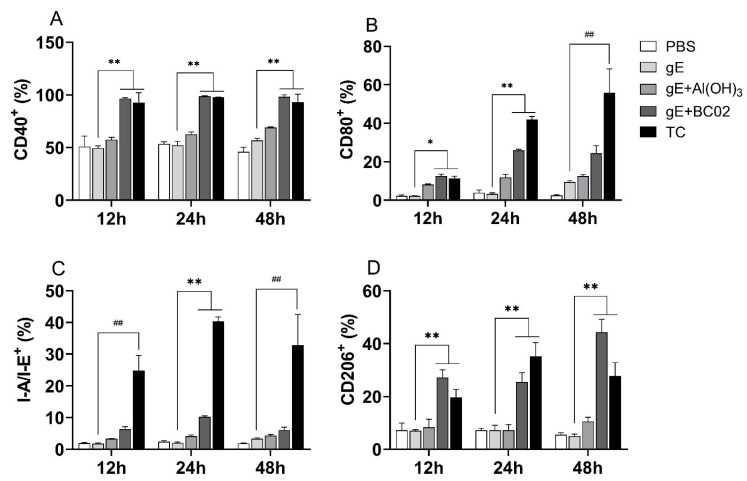
BC02-adjuvanted gE experimental vaccine up-regulates the expression of I-A/I-E and cell-surface costimulatory molecules in murine macrophages. (**A**–**D**): RAW264.7 cells were stimulated with 1/25 dose of experimental vaccine at different time points (12–48 h). The cells were collected and the abundance of costimulatory molecules CD40, CD80, I-A/I-E and CD206 on the cell surface was detected by flow cytometry. *, ** and ^##^ indicates that there is a significant statistical difference between 1/25 dose of TC and gE + BC02 MD compared with the gE group, or 1/25 dose of TC compared with gE group, respectively, *p* < 0.05.

The effect of 1/25 dose of the experimental vaccine on the expression of I-A/I-E, and cell-surface costimulatory molecules, such as CD40 and CD80, from murine macrophages were investigated by flow cytometry. The findings showed that 1/25 dose of MD and TC stimulation of macrophages increased the expression of CD40 and CD80 by a twofold factor at 12 h and 24 h, respectively, compared with the 1/25 dose of the non-adjuvanted gE experimental vaccine group (all *p* < 0.05, Figure 7A,B). Interestingly, the expression of I-A/I-E peaked at 24 h, followed by a gradual reduction until 48 h, and this trend was also consistent with the TC group (Figure 7C).

Further analysis of the abundance of CD206 in each stimulated cell showed that 1/25 dose of MD could induce a stronger expression of CD206 compared with the TC group, which was significantly higher than 1/25 dose of the non-adjuvanted gE experimental vaccine group (all *p* < 0.05, Figure 7D). These results suggest that the BC02-adjuvanted experimental vaccine can activate the immune response of macrophages and induce stronger M2-like macrophages by upregulating the expression of I-A/I-E and other costimulatory molecules on the cell surface.

### 3.6. Enhancement of Phagocytosis in RAW264.7 by the Experimental Vaccine with BC02

**Figure 8:**
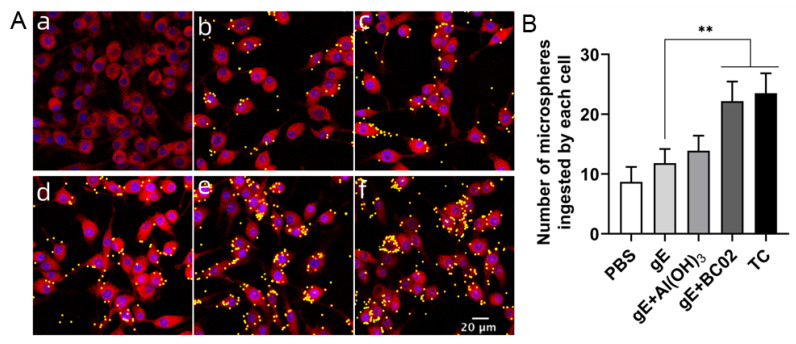
The BC02-adjuvanted gE experimental vaccine up-regulates the phagocytosis of murine macrophages. RAW264.7 cells were stimulated with 1/25 dose of experimental vaccine and incubated with fluorescent microspheres at 37 °C for 1 h. After washing with sterile PBS, macrophages were labeled with CellMask™ Deep Red plasma membrane stain, and confocal microscopy was used to analyze phagocytosis. (**A**): Representative images after laser scanning confocal microscopy acquisition showing differential phagocytosis by RAW264.7 cells. (**a**–**f**): for Blank control, PBS, 1/25 dose of gE, gE + Al(OH)_3_, gE + BC02 and TC groups, respectively. (**B**): Confocal microscopy assisted count of fluorescent microspheres internalized by individual cells. ** Indicates that there is a significant statistical difference between 1/25 dose of TC and gE + BC02 MD compared with the gE group; *p* < 0.05.

Using confocal microscopy, we counted the number of fluorescent microspheres engulfed by a single macrophage in the same field. The results showed that the average number of phagocytosed microspheres in the 1/25 dose of MD and TC groups was significantly higher than that in the non-adjuvanted gE experimental vaccine group (all *p* < 0.05, Figure 8B). Meanwhile, although the level of phagocytosis was also increased in the 1/25 dose of the Al(OH)_3_-adjuvanted gE experimental vaccine compared with the non-adjuvanted gE experimental vaccine group, the difference was not statistically significant (Figure 8B).

## 4. Discussion

Herpes zoster (HZ) is a neurocutaneous disease caused by the reactivation of VZV from a latent infection of dorsal sensory or cranial nerve ganglia after an earlier primary infection with VZV [19]. HZ is most common in people older than 60 years with age-related weakening of the immune system. It also occurs more often in immunocompromised people, including those receiving chemotherapy, radiotherapy or steroids as well as people with disease-related immunosuppression from HIV/AIDS, diabetes mellitus or cancer. In addition, having a family history of HZ and having varicella before the age of 1 year also increase the risk [19]. Currently, there are only two HZ vaccines approved for human use worldwide, Zostavax^®^ (Merck & Co., Inc., Kenilworth, NJ, USA) and Shingrix^®^ (Glaxo Smith Kline, Rockville, MD, USA). Zostavax^®^ is a zoster vaccine live (ZVL), which has been approved in the European Union since 2006 for the prevention of HZ and postherpetic neuralgia (PHN) in adults aged 50 years and older [20]. Shingrix^®^ is a recombinant zoster vaccine (RZV), which was developed from subunit zoster vaccine containing VZV glycoprotein E and the AS01_B_ adjuvant system. Based on its superior immunogenicity, efficacy and safety, Shingrix^®^ has been approved by the U.S. Food and Drug Administration (FDA) and recommended by the Advisory Committee on Immunization Practices (ACIP) as the preferred vaccine for the prevention of herpes zoster in immunocompetent adults aged 50 years and older [21,22,23].

As a major target of VZV-specific CD4^+^ T-cell responses, gE, in contrast to those of other alphaherpesviruses, is the most abundant glycoprotein in VZV virions and infected cells, which is essential for viral replication and cell-to-cell transmission [24,25,26]. The liposome-based AS01_B_ adjuvant system contains two immune stimulators. The first one is 3-O-desacyl-4 -monophosphoryl lipidA (MPL), which is a TLR4 agonist that stimulates NF-κB transcription and cytokine production and activates antigen-presenting cells (APCs). The second one is Quillaja saponaria Molina fraction 21 (QS21), which is a natural saponin that promotes antigen-specific antibodies and CD4^+^ T-cell responses [27,28]. Similarly, in this study, we also used VZV gE protein combined with BC02 compound adjuvant composed of the natural TLR9 agonist BCG CpG DNA as well as the traditional classical adjuvant Al(OH)_3_. Previous studies have shown that the BCG CpG DNA bio-adjuvant can rapidly activate innate immune response through the NF-κB and MAPK signaling pathways [5]. Similarly, the immunoenhancement effect of aluminum adjuvant lies in the amount of antigen adsorptive, its mainly in promoting the phagocytosis of APC, such as promoting the phagocytosis and processing of antigen by dendritic cells and macrophages [29]. Aluminum-carrying macrophages showed phenotypic and functional changes that were typical of osteomyelitis like dendritic cells and demonstrated the ability to induce MHC Class II antigen-specific memory responses [30]. In this study, we focused our evaluation on identifying the most immunogenic vaccine formulation with respect to CMI or humoral responses. The conducted immunogenicity studies were used to identify a potential candidate vaccine that contains different doses of the recombinant gE antigen using a prime-boosted mouse model.

The results of comparative experiments showed that the immunogenicity of gE, both cellular and humoral, was considerably increased if formulated with the BC02 adjuvant, while the unadjuvanted gE experimental vaccine was poorly immunogenic. Importantly, the BC02-based formulation was also shown to induce higher frequencies of CD4^+^ T cells producing IL-2 and IFN-γ compared with the evaluated Al(OH)_3_ formulations. Although there was no significant difference between the high (10 μg), medium (5 μg) and low (2.5 μg) doses of the experimental vaccine, and there was no related trend of significantly enhanced CMI response as the dose of gE glycoprotein increased, the compatibility effect of BC02 with low and medium doses of gE was better than that with high doses. This also emphasized the importance of the BC02 adjuvant for the quality of the induced CD4^+^ T cell responses. Fortunately, those are the desired outcomes, because the CMI response to VZV is currently recognized as the strongest immunological predictor of reduced HZ incidence and severity as well as reduced incidence of HZ-associated PHN in clinical investigations [31,32,33,34]. In addition, these results are also consistent with the effect of AS01B adjuvant combined with gE protein in the mouse model [35,36]. The VZV-specific antibodies are not thought to be essential to confer protection against HZ or HZ-associated PHN or the severity thereof [37,38,39]. However, as an important complement to the HZ vaccine response to CMI, humoral immunity studies have focused more on the concentration of VZV-specific antibodies, emphasizing the function and quality of IgG, such as avidity and neutralization. In terms of sensitivity, antibody neutralization tests typically detect antibody titers that are two- to four-fold higher than the FAMA titer [40]. However, those two experimental methods are effective for the specific determination of the humoral immunity status of VZV in healthy or immunosuppressed individuals, and they also have the effect of supporting each other. Therefore, in this work, we compared the immune response of VZV gE-specific antibodies in the Al(OH)_3_ or BC02-adjuvanted gE experimental vaccine as measured by a FAMA and plaque reduction test, and obtained satisfactory results.

Stimulating innate immune responses is a prerequisite to generate adaptive vaccine responses. Thus, the magnitude of local innate immune responses starting at the vaccine delivery site initially controls the subsequent adaptive immune responses. The induction of an efficient vaccine response requires some degree of local inflammation to trigger and support the sequence of immunological events that will lead to the adaptive immunity. Therefore, adjuvants administered to the muscle have a central role in the induction of transient inflammation at the delivery site, which promotes immune cell recruitment and activation. This inflammation is likely to lead to a better vaccine antigen uptake by critical infiltrating cell types and the migration of vaccine-loaded cells to the draining lymph nodes (dLNs) to establish the adaptive immunity. It is thus equally important to conduct in vitro stimulation experiments with a mouse monocyte-macrophage leukemia cell line RAW264.7, analyze the immunological indicators related to macrophage activation and evaluate the activation effect of the BC02-adjuvant gE experimental vaccine on innate immunity. Based on the immune doses obtained in previous adaptive immunity studies, we stimulated macrophages with 1/25 dose of the gE + BC02 MD for different time periods, and the results showed that 1/25 dose of MD could significantly induce the expression of TNF-α, MCP-1, IL-6 and GM-CSF, with effects similar to those of widely studied adjuvants (e.g., MF59, AS01, AS03 and AS04) [41,42,43,44,45] and another study on high levels of IL-6 secreted by vzv infected skin explants [46]. Meanwhile, flow cytometry revealed that I-A/I-E and co-stimulatory molecules, such as CD80, CD86 and CD40, were significantly upregulated in stimulated cells; these are key innate immune events for effective antigen presentation and priming of antigen-specific naive T cells. Using confocal microscopy, we found that the phagocytic function of stimulated cells was improved, which was significantly higher than that of the non-adjuvanted gE experimental vaccine group. These results are also consistent with the previously detected up-regulation of chemokines (pro-inflammatory cytokines) or costimulatory molecules.

In conclusion, the experimental HZ vaccine with BC02 adjuvant recombinant different doses of gE glycoprotein induces an Th1-based T cell immune response, and the favorable alternative dose was a moderate dose of gE antigen. In vivo experiments on mice showed that incorporating the BC02 compound adjuvant enhanced the quality and function of the VZV gE glycoprotein-induced humoral immune response, promoted antibody seroconversion and improved the antibody neutralization activity. Through in vitro experiments, we demonstrated the enhancement of cellular activation, antigen uptake and antigen presentation capacity due to innate activation by the BC02 adjuvant. The benefits of adding the BC02 adjuvant to the recombinant gE experimental vaccine were confirmed by the substantial improvement in the elicited immune response.

## Data Availability

Not applicable.
